# Gender Stereotypes and Sexual Double Standards Among Chilean University Students: Psychometric and Sociocultural Evidence

**DOI:** 10.3390/bs16020200

**Published:** 2026-01-30

**Authors:** Ximena Briceño-Olivera, Miguel Galván-Cabello, Julio Tereucán, Vicenta Rodriguez, Marisol Lemunao, Claudio Briceño

**Affiliations:** 1 Departamento de Trabajo Social, Universidad de La Frontera, Temuco 4780000, Chile; ximena.briceno@ufrontera.cl (X.B.-O.); miguel.galvan@ufrontera.cl (M.G.-C.); julio.tereucan@ufrontera.cl (J.T.); m.lemunao01@ufromail.cl (M.L.); 2 Facultad de Ciencias Sociales de Talavera de la Reina, Universidad de Castilla La Mancha, 02071 Albacete, Spain; vicenta.rodriguez@uclm.es

**Keywords:** gender stereotypes, sexual double standard, factor analysis, gender roles, gender moral norms, higher education

## Abstract

This study presents the results of the application of an instrument designed to assess gender stereotypes in a cohort of 672 Chilean students. An exploratory factor analysis identified three main dimensions: traditional masculinity, feminine caregiving stereotypes, and moralizing norms regarding female behavior. To further distinguish attitudinal variation, a segmented analytical approach is proposed. The results are discussed in light of gender theory and differentiated socialization, highlighting how these stereotypical configurations operate as a psychological and normative substrate that contributes to the persistence of double standards based on gender, legitimizing greater sexual freedom for men and stricter moral control over female sexuality. Finally, the educational implications of these findings are discussed, emphasizing the need for educational interventions that critically question gender stereotypes and promote higher education oriented toward equity and non-sexist training.

## 1. Introduction

Gender stereotypes are socially shared belief systems that assign different attributes, roles, and expectations to men and women (Guillén et al., 2024; Sobarzo et al., 2025), significantly influencing their behaviors, life trajectories, and social relationships. These representations are constructed early on through processes of differential socialization and are reinforced by key institutions such as the family, the media, and the education system (Navarro & Zaragoza, 2024). In university contexts, gender stereotypes can influence career choices, academic performance, leadership expectations, and forms of interpersonal relationships, reproducing persistent inequalities even in settings that discursively promote gender equality.

From a psychosocial perspective, gender stereotypes not only describe supposed differences between men and women, but also fulfill a normative and prescriptive function, defining what is considered acceptable, desirable, or morally legitimate for each gender. In this sense, various studies have pointed out that these beliefs operate as cognitive frameworks that organize the social evaluation of behaviors, particularly in the realm of sexuality, where gender norms tend to be applied asymmetrically.

One of the most persistent expressions of this asymmetry is the sexual double standard (SDS). According to Chadwick-Brown and Endendijk (2024), it is the differential evaluation of the sexual behavior of men and women, which legitimizes greater male sexual freedom and stricter moral control over female sexuality. The literature has shown that the SDS is not limited to explicit sexual attitudes, but is underpinned by broader belief systems about masculinity, femininity, and sexual morality, which function as psychological and cultural antecedents to these differential evaluations.

In line with the objectives of the present Special Issue, this study approaches the sexual double standard from an individual and ideological perspective, focusing on how gender stereotypes and moral norms operate as psychological foundations that legitimize differential evaluations of sexual behavior. Rather than measuring SDS directly as an attitudinal outcome, this research examines the belief systems that sustain it, contributing to the understanding of how sexual double standards are reproduced through gendered expectations surrounding masculinity, femininity, and sexual morality.

In this framework, gender stereotypes, especially those linked to hegemonic masculinity, nurturing femininity, and traditional moral norms, can be understood as a cognitive and normative substrate that contributes to the persistence of sexual double standards (Cervilla et al., 2024), even among younger generations socialized in contexts of greater normative openness. The naturalization of male hypersexuality, the association of feminine value with care and relational responsibility, and the symbolic sanctioning of female sexual transgression form a network of beliefs that legitimizes inequality in the evaluation of sexuality according to gender.

In Latin America, and particularly in Chile, various studies have shown the persistence of traditional gender stereotypes in universities, both among students and teachers, as well as their influence on teaching practices, academic expectations, and career paths. However, there is a need for psychometrically validated instruments that allow for the systematic evaluation of these beliefs and an understanding of their internal structure, as well as their relationship to broader sociocultural phenomena, such as double standard based on gender.

In this context, the present study aims to apply and psychometrically analyze the Scale of Attitudes toward Gender Roles in a sample of Chilean university students in order to identify the predominant dimensions of gender stereotypes and examine their relevance as psychological foundations of the sexual double standard. By placing the empirical findings within the conceptual framework of the sexual double standard, and specifically within the individual, relational, and ideological levels proposed in this Special Issue, this work seeks to contribute to the understanding of the sociocultural mechanisms that sustain gender inequality in the domain of sexuality, providing relevant evidence for the design of educational interventions aimed at promoting more equitable and non-sexist relationships in higher education.

### Gender Stereotypes and Traditional Roles

The gender perspective serves as an analytical tool for understanding how the differences attributed to men and women are not merely biological, but rather socially and culturally constructed, generating structural inequalities across multiple life domains. This approach recognizes that gender relations are mediated by systems of power that assign differentiated roles, hierarchies, and opportunities, directly affecting access to rights, resources, and decision-making spaces (Scott, 1990; Guillén et al., 2024; Sobarzo et al., 2025).

From this perspective, it is essential to examine the symbolic and normative mechanisms that sustain such inequalities, including gender stereotypes in particular. These stereotypes act as collective representations and define what is considered “appropriate” or “natural” for each gender, therefore conditioning people’s opportunities and personal development (Scott, 1990; Sobarzo et al., 2025). In this regard, Butler (1990) argues that gender is not a stable essence, but a performative construction reproduced through everyday practices, reinforcing differentiated roles that limit individual agency. This production takes place across various social institutions–such as the family, the media, and, in particular, the education system–which function as key spaces for the transmission and legitimization of gender roles (Connell, 1995). In the Latin American context, both Lamas (2000) and Araya and Guizardi (2025) emphasize that these institutions contribute to the naturalization of inequalities by presenting the differences between men and women as inevitable or biologically determined, thereby obscuring their cultural and political nature.

In the Chilean context, studies such as those by Arcos et al. (2006) and Cortés et al. (2025) have shown that both students and teachers maintain traditional views and possess limited knowledge about gender, which facilitates the reproduction of socialization models that perpetuate inequality in the university setting.

Gender stereotypes structure how men and women are perceived, valued, and assigned social functions, shaping their opportunities, behaviors, and life trajectories. These socially shared beliefs attribute qualities such as rationality, strength, and authority to men, whereas women are associated with characteristics such as emotionality, tenderness, and dependence (López-Sáez et al., 2008). Although some of these stereotypes may appear positive or harmless–such as considering women more empathetic or men more decisive–they operate as mechanisms of symbolic control that reinforce the established social order (Pérez-García et al., 2025; Polanco Cerón & Morrison, 2024; Merchan & Rendon, 2025).

This form of control is not exercised coercively, but rather through the cultural norms, media representations, social expectations, and even educational practices that define what is considered “appropriate” or “normal” for each gender. As Butler (1990) argues, gender operates as a performative construction that is continually reaffirmed through social and discursive practices, thereby restricting possible forms of subjectivity. In a similar vein, Bourdieu (2000) notes that such schemas act as mechanisms of symbolic domination– invisible yet highly effective in reproducing hierarchies.

Thus, stereotypes impose limits that constrain individuals’ personal and professional expression, penalizing deviations from the dominant model. An example of this is the difficulty faced by women who assume leadership positions, as they are often perceived as authoritarian or lacking empathy, in contrast to the expected congruence between the female gender and caregiving, as described in Eagly and Karau’s (2002) role congruity theory. Likewise, men who express emotions or engage in caregiving tasks are socially sanctioned for transgressing the ideal of hegemonic masculinity (Connell, 1995). These sanctions operate as what Foucault (1975) referred to as disciplinary instruments: devices that promote self-regulation and conformity to established norms. Thus, gender stereotypes not only describe supposed differences but also prescribe limits that perpetuate hierarchical power relations between men and women.

In the case of women, these stereotypes have a direct impact on their personal, academic, and professional development opportunities. For example, by being perceived as naturally more suited for caregiving, docility, or domestic life, many women face social pressures that push them toward certain roles (mother, wife, caregiver) and discourage their participation in traditionally masculinized spaces such as science, politics, or leadership positions.

This unequal distribution of expectations limits women’s freedom of choice and generates both external and internal barriers. The external barriers include their underrepresentation in strategic fields such as science, politics, and technology, as well as the lower credibility they often face in male-dominated professional environments (Eagly & Karau, 2002; UNESCO, 2017). Internal barriers include self-exclusion rooted in doubts about their own abilities, a phenomenon widely described as impostor syndrome, which disproportionately affects women in highly demanding academic and professional settings (Clance & Imes, 1978; Cokley et al., 2018).

Furthermore, when women challenge traditional gender norms—by assuming leadership roles, displaying ambition, or prioritizing professional development—they are often judged more harshly than their male peers and face implicit social sanctions such as disqualification, isolation, or distrust (Heilman & Okimoto, 2007; Rudman & Phelan, 2008).

Hence, gender stereotypes not only reproduce a reductionist view of femininity, but also actively restrict women’s access to equitable conditions of recognition, autonomy, and growth, both in the private and public spheres. As Bourdieu (2000) warns, these dynamics represent forms of symbolic domination that appear “natural” but in reality reinforce a hierarchical model, conditioning women’s full development on conformity to traditional ideals and perpetuating structural inequalities under the guise of normalized roles.

In addition to describing how genders are perceived, these stereotypes are also prescriptive, as they indicate how individuals should behave according to their assigned sex (Castillo-Mayén & Montes-Berges, 2014a). This prescriptive nature manifests as mechanisms of punishment or social disapproval when an individual transgresses established gender norms, thereby perpetuating both overt and subtle forms of discrimination.

Authors such as Connell (1995), Umrath (2024) contribute to critical gender theory analysis by presenting the concept of hegemonic masculinity, defined as a cultural ideal that hierarchically categorizes various expressions of masculinity according to power, authority, and the subjugation of other genders. In contrast, femininity is often constructed as relational, passive, and oriented toward caregiving, which limits its recognition as autonomous. This dichotomy is reinforced from childhood through processes of differential socialization within the family, school, media, and other institutions.

Similarly, qualitative research has shown how institutional advertising and mass media messages continue to promote traditional gender models, even as discourses that challenge these norms are also emerging. Farías and Cuello (2018) analyze how university students navigate contradictory representations: on the one hand, they are exposed to gender equality discourses; on the other, they experience pressure to conform to traditional forms of masculinity or femininity. This generates identity tensions that reflect the dynamic and adaptive nature of gender stereotypes.

Additionally, the current literature indicates that stereotypes also intersect with other social variables such as class, ethnicity, or sexual orientation, amplifying inequalities. For example, women from low-income sectors may encounter a double burden: being regarded as less competent due to both their gender and their socioeconomic status. This intersectionality, proposed by Crenshaw (1989), is essential for understanding the complexity of stereotypes in real-world contexts.

For these reasons, addressing gender stereotypes in education cannot be limited to isolated awareness-raising actions; it requires a structural approach capable of transforming curricula, institutional practices, and teacher training. Assessing and monitoring these stereotypes using valid and culturally relevant instruments makes it possible to identify resistance, progress, and contradictions, thus aiding in the formulation of more inclusive and effective public policies. This is particularly important given that, in the educational sphere, gender stereotypes have significant effects on performance, academic self-efficacy, career choice, and interactions between teachers and students. Studies conducted in Chile show that these stereotypes not only persist but also directly influence pedagogical practices and perceptions of competence among both students and teachers.

For example, Espinoza and Albornoz (2023) found that both university professors and students reproduce stereotypical beliefs that influence how they value effort, intelligence, and academic authority according to gender. Along these lines, the study by Arcos et al. (2006) on gender perspectives among students and faculty at the Universidad Austral de Chile identified a predominantly undefined position regarding knowledge and opinions about gender, as well as the persistence of traditional views among academic staff. 

The authors showed through interviews and focus groups that, in the absence of the systematic incorporation of a gender perspective into institutional policies and educational processes, institutions are likely to perpetuate and legitimize traditional socialization patterns, thereby reinforcing structural inequalities. These findings complement those reported by Espinoza and Albornoz (2023), who show that teachers’ beliefs and practices, when not critically examined, can perpetuate gender biases and stereotypes in the university classroom.

From a historical perspective, Beauvoir (1949) pointed out that women are not born with a feminine essence, but are “constructed” through social processes that position them as “the other” in relation to the masculine, assigning them characteristics that limit their autonomy. In a similar vein, Bem (1981) proposed that gender schemas function as cognitive filters that organize information about what is considered appropriate for men and women, which explains the resistance to change even in contexts of apparent modernization of gender roles. Butler (1990), arguing from the perspective of performativity theory posits that gender identities are produced and reproduced through repetitive practices that consolidate cultural norms, evident in how stereotypes, even when partially transformed, continue to govern expected behavior. Thus, the study by Castillo-Mayén and Montes-Berges (2014b) confirms that gender stereotypes not only describe differences but also operate as normative frameworks that perpetuate inequalities, incorporating “modernized” traits without dismantling the hierarchies that sustain them.

Sexism operates in higher education as a psychosocial system that sustains discriminatory practices and attitudes based on gender norms, affecting students’ learning, identity, self-esteem, and life trajectories. According to Espinoza and Albornoz (2023), both university students and faculty reproduce explicit gender stereotypes and sexist pedagogical practices, particularly among those who place greater importance on masculinity. Moreover, for women, but not for men, identification with femininity is positively associated with academic self-concept, while the gender stereotypes held by instructors predict the adoption of sexist practices in the classroom. These findings demonstrate that sexism is not limited to individual prejudices but is rooted in institutional dynamics that shape attitudes to learning, academic achievement, and interactions in the educational process.

University socialization processes also shape young people’s expectations regarding the roles they will assume in the future: in the workplace, in the family, and in domestic life. In this regard, the study by Abarca et al. (2011) explored these expectations in a sample of 522 students in Santiago, Chile, using an adapted version of the Life Role Salience Scales (LRSS) and measuring participants’ degree of value conservatism. The results showed that men placed greater importance on work and marital roles than women, while value conservatism was a significant predictor for expectations related to marriage, parenthood, and household care, but not for the work role. This evidence indicates that, even before entering the professional world, gender differences and ideological convictions influence students’ visions of the future, reinforcing traditional patterns that may affect women’s academic and professional trajectories. Integrating these expectations into the analysis of sexism in higher education reveals how gender stereotypes are projected and consolidated in personal life plans, thereby perpetuating structural inequalities in educational and labor contexts.

Several studies have developed scales to measure attitudes toward gender roles, such as the Bem Sex Role Inventory (Bem, 1981) or the Gender Role Attitudes Scale (Pérez Sánchez et al., 2021). In the Latin American context, Cárdenas et al. (2010) and Gómez-Campos et al. (2023) have proposed cultural adaptations that capture the sociocultural specificities of the region.

The gender role scale used as the basis for this article was validated in Mexico in 2015 across several cultural contexts (Saldívar-Garduño et al., 2015a). The scale was consistent, obtaining a Cronbach’s alpha of 0.89 for its 18 items, which together explained 50.8% of the total variance and were grouped into three factors.

In 2024, findings from Guatemala showed that men exhibited significantly greater agreement with masculine roles (Me = 7.50) than women (Me = 6), U = 121, *p* = 0.012. In Colombia, Muñoz-Muñoz and Zambrano-Guerrero (2024) identified a three-factor structure explaining 50.8% of the common variance. In terms of reliability, Cronbach’s alpha was 0.89 for the total scale, and 0.78, 0.80, and 0.76 for each subscale. The results indicate that most participants do not hold a stereotypical perception of gender roles, although 47.4% of the sample does. Additionally, men were found to have more stereotypical perceptions than women. The study also revealed that perceptions of gender roles vary according to university faculty.

These tools, typically based on Likert-type scales, require statistical validation through factor analyses that confirm the internal coherence of their factors and their theoretical relevance. The use of such instruments in school settings contributes not only to diagnosis but also to the design of educational strategies informed by a gender perspective.

## 2. Materials and Methods

### 2.1. Study Design

This study is framed within an instrumental quantitative design aimed at assessing the psychometric properties of the Gender Role Attitudes Scale in a Chilean university population. The study seeks to provide empirical evidence on the structural validity and internal consistency of the instrument within the Chilean sociocultural context, thus contributing to the rigorous measurement of attitudes toward stereotyped gender roles among young populations.

### 2.2. Study Participants

To analyze the psychometric properties of the Gender Stereotype Scale, we recruited 711 university students from Chilean higher education institutions. Of these, 672 participants provided complete responses and signed informed consent (completion rate = 94.5%), and this complete-case sample was used for the main analyses. Data were gathered across institutions in four macro-zones, North, Central, South, and Austral South, selected for territorial representativeness and enrollment volume. With regard to the socioeconomic status of participants, 60.3% of the sample self-identifies as middle class. 21.6% identify as lower middle class and 15.5% as upper middle class. With regard to the upper and lower classes, 1.0% and 1.6% of participants respectively identify as such.

Participants were between 18 and 48 years old, with a mean age of 22.5 years (SD = 4.39), and the majority identified as women (57.3%). The only inclusion criterion was being enrolled as a regular student in a Chilean university at the time of the study. The resulting sample meets methodological requirements for conducting factor analyses, both in terms of size and the ratio of cases per item (Bentler & Chou, 1984; Bollen, 1989; Nunnally & Bernstein, 1995). Table 1 presents the characteristics of the participants.

### 2.3. Instruments

Identification and Family Socioeconomic Background Questionnaire: This instrument was developed to support the initial identification of participants. It consists of closed-ended questions that enable filtering and comparison with the scales applied later. The questions covered the following categories: sex, gender, age, geographic area of residence, and socioeconomic group.

Gender Role Scale: This instrument assesses attitudes toward traditional and equitable gender roles in different social contexts. It was originally developed by Saldívar-Garduño et al. (2015b) and comprises 18 items distributed across three factors: (1) Stereotypical Masculine Roles, (2) Stereotypical Feminine Roles, and (3) Traditional Roles. The scale uses a four-point Likert response format ranging from “Strongly disagree” to “Strongly agree,” identifying varying levels of agreement with normative statements about the roles of women and men in family, educational, and workplace contexts.

Embodied Cultural Capital Scale: This scale was designed by Díaz-Herrera et al. (2020) and is based on the theoretical dimensions of Bourdieu’s internalized cultural capital. It consists of 5 factors and 16 items measured using a 4-dimensional Likert scale ranging from “strongly agree” to “strongly disagree.” In this way, a classification of internalized cultural capital is established ranging from the minimum to maximum score, which we can qualitatively classify into internalized cultural capital of “conservatism” (highest score), “convergence” (medium score), and “reformism” (lowest score).

Ethnic Identity Scale (EIS): This scale was originally developed by Umaña-Taylor et al. (2004) and aims to assess the degree of identity with a particular ethnic group, as well as associated attitudes and behaviors. The EIS consists of 9 self-administered items on a four-point ordinal scale (1 = strongly disagree, 4 = strongly agree). It has three factors: exploration, resolution, and affirmation. Its factor structure has been evaluated in various populations (Yetter & Foutch, 2013; Yoon, 2011), and evidence of reliability and validity reports adequate indices of psychometric quality for its use (Umaña-Taylor et al., 2004).

### 2.4. Procedure

The study was conducted in three phases:

1. First, the original version of the Gender Role Attitudes Scale proposed by Saldívar-Garduño et al. (2015a) was reviewed, and its suitability for application to a Chilean university population was evaluated. Although the scale had already been validated in Spanish, a linguistic and contextual review was carried out by a team of researchers trained in gender studies, psychometrics, and sociocultural studies in Chile. The original wording of the items–comprising normative statements about the roles of women and men in family, educational, and work contexts, rated on a four-point Likert scale–was retained. Back-translation was not necessary, as the instrument was already in the target language.
2. For data collection, nine universities from different regions in Chile were contacted. Once the corresponding institutional authorization was obtained, administration of the questionnaire was coordinated through the QuestionPro platform. Dissemination was carried out via institutional email channels and the social media platforms of undergraduate programs. Students who agreed to participate signed a digital informed consent form, ensuring adherence to the ethical principles of the study. The protocol was approved by the Scientific Ethics Committee of the Universidad de La Frontera (protocol code N°089/24, approved on 12 June 2024). The estimated response time was approximately 15 min.
3. Finally, the psychometric properties of the scale were analyzed using the Chilean sample of 672 university students. Exploratory and confirmatory factor analyses were conducted, along with reliability and validity tests.

### 2.5. Data Analysis Plan

To assess the factorial structure and psychometric properties of the Gender Role Scale, a database was created using SPSS v22. For the cross-validation process, cases were randomly partitioned into two subsamples of equivalent size: one for model estimation and the other for model validation (Lloret-Segura et al., 2014).

In the exploratory subsample (n = 336), an exploratory factor analysis (EFA) was conducted using a polychoric correlation matrix due to the ordinal nature of the responses. For factor extraction, the unweighted least squares (ULS) method with PROMIN rotation was applied, and Horn’s parallel analysis was used as the criterion for determining the optimal number of factors to retain (Timmerman & Lorenzo-Seva, 2011). This methodological decision is supported by evidence demonstrating the superiority of using polychoric correlations and robust estimation methods when working with Likert-type scales (Freiberg et al., 2013). The EFA was performed using the FACTOR software v. 10.3.1 (Lorenzo-Seva & Ferrando, 2006).

Subsequently, a confirmatory factor analysis (CFA) was conducted with the confirmation subsample (n = 336) using the R language. Version 4.1; 2025 (R Core Team, 2025) and the lavaan package (Rosseel, 2012). As in the exploratory phase, the CFA was based on a polychoric correlation matrix and used the WLSMV estimation method, given its ability to yield accurate estimates in moderate samples and under simple factorial structures (Flora & Curran, 2004; Lloret-Segura et al., 2014). Different theoretical models reported in the international literature on attitudes toward gender roles were tested. The selection of the most appropriate model was based on the analysis of global fit indices: Satorra-Bentler chi-square, CFI, TLI, SRMR, and RMSEA. Indicators of acceptable fit were defined as CFI and TLI values equal to or greater than 0.90 (preferably ≥ 0.95), SRMR ≤ 0.08, and RMSEA ≤ 0.08 (Browne & Cudeck, 1993).

The internal consistency of the scale was evaluated using the ordinal alpha coefficient (Elosua & Zumbo, 2008), both for each dimension and for the total scale. Likewise, convergent and discriminant validity were evidenced through correlation analyses with other measures, in line with findings from previous research. Finally, to explore differences in total scale scores and its dimensions according to sex, Mann–Whitney U tests were applied.

## 3. Results

### 3.1. Exploratory Factor Analysis

Horn’s parallel analysis indicated the retention of three factors, as the observed eigenvalues exceeded the randomly generated eigenvalues. Sample adequacy indices demonstrated the suitability of conducting the EFA: KMO = [0.926] and Bartlett’s test of sphericity (χ^2^ = 10,849.474, df = 153, *p* < 0.001).

The resulting solution showed three correlated factors that together explained 66.9% of the total variance. Factor 1, Stereotypical Masculine Roles, explained 28.2%; Factor 2, Comparative Gender Stereotypes, explained 20.8%; and Factor 3, Traditional Roles in Family/Partner Contexts, explained 17.9%. Factor loadings ranged from 0.997 to 0.463, which are adequate values to support the retention of all items. Table 2 shows the distribution of items for each factor.

### 3.2. Confirmatory Factor Analysis (CFA)

Using the confirmation subsample (n = 336), a measurement model was estimated through SEM (Likert-type items treated as ordinal, WLSMV estimator) to test three specifications: (a) a two-factor correlated model (based on the theoretical proposal of the Gender Role Scale for the central region of Mexico); (b) a three-factor model (derived from the EFA conducted in this study); and (c) a four-factor model proposed by Saldívar-Garduño et al. (2015a) for the northern region of Mexico.

In terms of model fit, the three-factor model showed the best fit indices (Table 3), outperforming the other two models under evaluation. None of the modification indices exceeded a threshold that would justify freeing residual covariances. To preserve theoretical integrity and given the goodness-of-fit indicators, the three correlated-factor model was retained.

### 3.3. Reliability Analysis of the Scale

Given the ordinal nature of the data, the ordinal alpha coefficient was used (Elosua & Zumbo, 2008). The results indicated values of 0.83 for the factor Stereotypical Masculine Roles, 0.87 for the factor Stereotypical Feminine Roles, and 0.86 for the factor Traditional Roles. For the total scale, a value of 0.93 was obtained. These results are considered adequate according to the cutoff criteria suggested by the specialized literature.

### 3.4. Discriminant and Convergent Validity

Spearman correlation results show that the Gender Role Scale is not associated in a statistically significant way with the Ethnic Identity Scale (ρ = −0.056, *p* = 0.548), supporting discriminant validity. By contrast, the Gender Role Scale correlates significantly with the Embodied Cultural Capital Scale (ρ = −0.667, *p* < 0.001). Consistent with a Bourdieusian view, embodied cultural capital reflects gendered appearance norms. Under our scoring, where higher values reflect greater sexism/stereotypy, the negative association indicates that higher levels of embodied cultural capital (as operationalized here) are related to lower endorsement of sexist stereotypes, providing convergent validity in the expected sense for opposite poles (Robinovich et al., 2018).

To complement our convergent validity assessment, we computed the average variance extracted (AVE) for each latent factor, using the criterion that AVE ≥ 0.50 indicates a factor explains more than half of its items’ variance. The AVE values were Stereotypical Masculine Roles = 0.549; Stereotypical Feminine Roles = 0.630 and Traditional Roles = 0.620, confirming that all three factors demonstrate strong convergent validity.

### 3.5. Comparisons of Gender Role Attitude Scale Scores by Sex and Age

Concerning the comparisons of scale scores by sex, the Mann–Whitney U test was used. Significant differences were observed in two of the three dimensions, as well as in the total score. Men (n = 287) showed greater endorsement of stereotypical masculine roles than women. They also reported higher scores on traditional roles and on the total scale score.

In terms of agreement with stereotypical feminine roles, no statistically significant difference was found between men and women (*M* = 2.02, *SD* = 0.62 vs. *M* = 1.81, *SD* = 0.62), *Z* = −0.838, *p* = 0.402.

Taken together, the results indicate greater acceptance of masculine stereotypes and traditional roles among men, while adherence to feminine stereotypes is similar across sexes. Table 4 presents comparisons of each factor by gender.

## 4. Discussion

The central objective of this study was to evaluate the psychometric properties of the Gender Role Attitudes Scale in a Chilean university population using an instrumental design that integrated exploratory and confirmatory factor analyses in a large and territorially diverse sample. The results obtained confirm that the applied version of the instrument shows adequate levels of structural validity and internal consistency, supporting its relevance for the study of gender attitudes in Latin American sociocultural contexts. The replication of a three-factor structure (stereotypical masculine roles, stereotypical feminine roles, and traditional roles) suggests that, despite discursive changes surrounding gender equality over the last decade, dichotomous schemas that assign differentiated responsibilities to women and men continue to constitute a meaningful frame of reference among young adults.

It is important to note that the present study measures gender stereotypes rather than explicit sexist attitudes. While these constructs are theoretically related, stereotypes do not necessarily imply conscious endorsement of sexism. Therefore, the interpretations offered here should be understood as indicative of potential symbolic and normative mechanisms, rather than as direct evidence of sexist intent or domination.

Beyond the psychometric indicators, the findings make it possible to problematize how beliefs about gender roles are maintained or transformed among generations socialized in contexts of greater normative openness, yet still embedded in culturally conservative structures. The differences observed by sex–higher levels of endorsement of masculine stereotypes and traditional roles among men, and greater ambivalence among women–reflect an ongoing cultural transition in which gender equality is assumed to be a desirable value but does not necessarily translate into a critical examination of internalized gender mandates. In this regard, the results align with research highlighting the coexistence of egalitarian discourses with subtle forms of resistance to change in educational and family contexts (Glick & Fiske, 1996; Levant & Wong, 2017), reinforcing the need to continue developing instruments sensitive to these emerging ideological tensions. This study also allows us to explicitly interpret gender stereotypes as psychological and moral foundations that underpin sexual double standard (SDS) among Chilean university students. Rather than operating as isolated beliefs, the dimensions identified through factor analysis reflect an interrelated system of norms that regulates the sexuality of men and women asymmetrically. In this sense, SDS emerges not only as an attitudinal bias regarding sexual behavior, but also as the result of deeply internalized gender expectations around masculinity, femininity, and moral value.

From the perspective proposed in this Special Issue, these findings contribute to the understanding of the sexual double standard at multiple levels. At the individual level, they reveal internalized beliefs about gender and sexuality; at the relational level, they reflect moral expectations linked to fidelity, care, and intimate relationships; and at the ideological level, they expose gendered moral norms that legitimize asymmetrical sexual evaluations.

Accordingly, the following analysis interprets each identified factor as a specific mechanism through which the sexual double standard is reproduced. Rather than treating gender stereotypes as isolated attitudes, the discussion examines how hegemonic masculinity, caregiving femininity, and gendered sexual morality interact to sustain differential standards of sexual behavior.

### 4.1. Identified Factors

The factor analysis revealed three main factors:

Factor 1: Traditional Masculinity and Hegemony

This factor groups together statements that reinforce attributes of men as dominant, emotionally restrained, and sexually active. The high loading of items such as “A real man does NOT show his feelings” (0.937) and “A man needs several sexual partners” (0.919) reflects the persistence of the ideal of hegemonic masculinity (Connell, 1995), understood as a cultural model that legitimizes male superiority over women and over other forms of masculinity. This ideal prescribes power, authority, and self-sufficiency as “natural” attributes in men.

From Butler’s (1990) theory of performativity, these discourses persist because they are repeated and naturalized through everyday practice, disciplining young men to inhibit vulnerability or emotional expression. At a symbolic level, these mandates operate as described by Bourdieu (2000): invisible mechanisms of domination that act from within subjects, regulating their behaviors without the need for explicit coercion.

This pattern is consistent with what Kimmel (2006) refers to as toxic masculinity, in which male toughness, emotional detachment, and hypersexuality are valued, whereas tenderness, emotional dependence, or monogamy are interpreted as signs of weakness.

This factor not only reproduces gender inequalities, but also restricts male subjectivity by preventing the full development of young men’s emotional and relational lives, affecting their psychological well-being and the quality of their relationships, and reinforces beliefs that portray men as emotionally restrained, dominant, and sexually free. Items such as “A man needs multiple sexual partners” or “Men are unfaithful by nature” directly reflect a cultural logic that conceives of male sexual activity as natural and socially acceptable. This configuration is linked to the concept of hegemonic masculinity proposed by Connell (1995), which legitimizes male authority and autonomy, naturalizing behaviors that would be morally sanctioned if performed by women.

And from the perspective of sexual double standard, this factor contributes to the normalization of male sexual freedom and the minimization of men’s moral responsibility for their sexual behavior. Sexual exploration, multiple partners, and infidelity are presented as inherent traits of masculinity, rather than behaviors subject to ethical evaluation. As Bourdieu (2000) argues, these beliefs operate as forms of symbolic violence, acting invisibly but effectively, shaping dispositions and expectations without the need for explicit coercion. In this way, male sexuality is positioned as autonomous and expansive, reinforcing a moral asymmetry central to the SDS.

Factor 2: Feminine Stereotypes and Caregiving Roles

This factor groups items that associate women with supposedly innate characteristics such as caregiving, emotionality, and the ability to attend to others. Examples such as “A mother is more affectionate than a father” (0.829) or “Women have a greater ability to care for the sick” (0.677) refer to an essentialization of femininity–namely, the idea that women possess “natural” qualities for child-rearing and domestic tasks (Gilligan, 1982).

From the perspective of role congruity theory (Eagly & Karau, 2002), these stereotypes create a mismatch between social expectations of femininity—centered on caregiving—and aspirations for autonomy or leadership. This helps explain why women who enter positions of power are often perceived as “authoritarian” or “cold.” These findings reinforce what Lagarde (1990) argues: the cultural construction of subordinated femininity, the worth of which is measured in terms of self-sacrifice and devotion to others.

Moreover, the reproduction of these stereotypes in the educational sphere aligns with Lamas (2000), who shows how institutions naturalize inequalities by presenting the sexual division of labor as inevitable.

Although these stereotypes may appear benevolent, they constrain female agency by confining women’s social value to caregiving roles, thereby reinforcing structural barriers to their full participation in academic, professional, and political domains. Rather than functioning as genuinely positive attributes, such traits operate as prescriptive norms that evaluate women primarily in terms of devotion, emotional labor, and self-sacrifice. This configuration contributes indirectly but profoundly to sexual double standard by establishing that female sexuality must be consistent with values of relational commitment and moral responsibility. When femininity is defined in terms of care and devotion, women’s sexual autonomy is restricted. As Lagarde (1990) points out, in Latin American contexts, the cultural construction of femininity links the value of women to the preservation of bonds, family, and emotional cohesion, limiting their sexual agency.

In this framework, transgressions of relational norms such as sexual autonomy or non-monogamy tend to be interpreted as moral failings rather than legitimate expressions of individual freedom. Thus, even in the absence of explicit sexual judgments, stereotypes of care form a moral backdrop that supports gender-differentiated evaluations of sexual behavior.

The absence of statistically significant gender differences in the endorsement of feminine stereotypes deserves particular attention. One possible explanation is the strong cultural naturalization of caregiving roles, which may lead both men and women to internalize these beliefs as socially desirable or morally positive. From a psychosocial perspective, this shared endorsement may reflect the persistence of benevolent gender norms that are less likely to be questioned, even in contexts of increasing gender equality discourses.

Additionally, women’s endorsement of caregiving stereotypes may be understood as a form of adaptive identification with socially valued roles, particularly in cultural contexts where care continues to be a central source of feminine recognition. These interpretations suggest that the persistence of feminine stereotypes is not solely imposed externally, but may also be reproduced through internalized norms and identity processes.

Factor 3: Gender Norms and Sexual Morality

This factor includes statements such as “Infidelity is unforgivable in a woman” (0.462) and “A good woman should take care of her partner” (0.518), which reinforce a gender-differentiated moral code. While male infidelity is often tolerated or even normalized, female infidelity is severely sanctioned. Similarly, women are expected to display obedience and attentiveness, subordinating their autonomy to relational demands.

These findings illustrate what Lagarde (1990) and Jerez and Alemán (2024) described as the cultural construction of femininity around submission, and they align with Bourdieu’s (2000) notion of symbolic violence, insofar as a double sexual standard is legitimized under the guise of unquestionable social norms. Likewise, these types of prescriptions operate as what Foucault (1975) described: disciplinary apparatuses that regulate bodies and behaviors, promoting feminine self-regulation. From Crenshaw’s intersectional theory (1989), it can be pointed out that these norms do not impact all women homogeneously, but rather intensify according to conditions of class, ethnicity or sexuality.

This factor reveals the persistence of a double sexual and emotional standard that limits women’s autonomy, legitimizing deeper inequalities and creating a symbolic terrain that can sustain practices of discrimination and gender-based violence.

### 4.2. Comparison with Previous Studies

The results partially align with previous studies in the Latin American context (Cárdenas et al., 2010; Gómez-Campos et al., 2023), although the factors identified here reveal a structure more centered on moral and emotional dimensions, which may reflect particular characteristics in the Chilean context.

Building on these results, it is essential to interpret the theoretical, educational, and sociocultural implications of the stereotypes identified. The coexistence of traditional discourses and emerging values of equality suggests that gender beliefs among Chilean university students are in a process of cultural transition, where traditional socialization frameworks coexist with new ways of understanding gender identities.

Within this framework, the following discussion seeks to articulate the empirical findings with the main theoretical contributions reviewed, examining how the dimensions of hegemonic masculinity, caring femininity, and differentiated sexual morality reflect the persistence of symbolic power structures, but also the emergence of processes of change in the educational sphere. This analysis aims to situate the results within a psychosocial and cultural approach to behavior, providing evidence on how gender stereotypes continue to shape attitudes, practices, and development opportunities in Chilean higher education.

Taken together, these moral prescriptions legitimize a social order in which female sexuality is strictly regulated, while male sexuality remains relatively free from control. This asymmetry constitutes the core of the sexual double standard, sustained not only by explicit sexual attitudes, but also by broader systems of gender stereotypes and moral expectations.

## 5. Conclusions

The results of this study indicate that gender stereotypes are still present among Chilean university students, although their manifestation is neither unidimensional nor homogeneous. The identification of three differentiated factors–traditional and hegemonic masculinity, feminine stereotypes associated with caregiving, and gender norms linked to sexual morality–demonstrates that sexist beliefs operate through complementary dimensions that reinforce both male domination and female subordination.

Although this study does not include a direct measure of adherence to the sexual double standard, the results provide empirical evidence on the psychological and ideological foundations that sustain SDS, in line with individual, relational, and ideological levels.

This finding is consistent with Connell’s (1995) notion of hegemonic masculinity, insofar as it reveals a naturalization of attributes such as emotional hardness, male hypersexuality, and authority over the feminine. Likewise, the second and third factors reflect what Lagarde (1990) describes as the cultural construction of femininity around caregiving and obedience, where women’s worth is measured by their ability to care for others and conform to restrictive moral norms.

A central aspect of the results is that the stereotypes identified are not perceived uniformly; rather, they may be expressed through diverse profiles. While some students consistently reproduce conventional discourses, others show partial resistance or explicit rejection of these stereotypes.

This heterogeneity is consistent with the findings of Espinoza and Albornoz (2023), who reported that both instructors and students maintain gender stereotypes, although with nuances that shape their perceptions of competence and their pedagogical practices. Likewise, it aligns with what was reported by Arcos et al. (2006) at the Universidad Austral de Chile, where limited knowledge and the persistence of traditional positions were observed, reinforcing the idea that higher education institutions–consciously or not–reproduce sexist discourses.

This finding suggests that stereotypes do not operate solely as rigid structures, but rather form part of a dynamic and contested field in which resistance, adaptation, and transformation coexist. In line with social role theory (Eagly, 1987), social changes in the roles performed by women and men can be interpreted as influencing the partial reconfiguration of stereotypes, although without fully eliminating their normative and prescriptive foundations.

From an educational perspective, these results underscore the need to design differentiated interventions that acknowledge the diversity of attitudes among the student body. The existence of varied profiles indicates that promoting general discourses of equality is insufficient; rather, pedagogical strategies are required that enable critical reflection on hegemonic masculinity, challenge the naturalization of caregiving as a feminine function, and deconstruct the moral norms that limit women’s sexual autonomy. The instrument used in this study emerges as a valuable diagnostic tool, both for research and for the implementation of university policies aimed at gender equity.

In summary, the findings show that gender stereotypes persist in Chilean higher education, but their expression is marked by contradictions and processes of change. Recognizing these tensions not only allows for a better understanding of the current state of gender beliefs among students, but also opens opportunities to strengthen critical and non-sexist education that contributes to cultural transformation toward a more equitable society. From the perspective of sexual double standard, the findings of this study highlight that SDS among Chilean university students is not based solely on explicit sexual attitudes, but on a broader psychological and moral infrastructure composed of deeply rooted gender stereotypes. The coexistence of hegemonic masculinity, femininity associated with caregiving, and gender-differentiated moral norms creates fertile ground for the unequal evaluation of sexual behavior, legitimizing greater sexual freedom for men while imposing stricter moral control on women. These results suggest that the persistence of sexual double standards among young adults is anchored in internalized beliefs about gender, morality, and relational value, which operate as subtle but powerful mechanisms for reproducing inequality, even in contexts characterized by discourses of greater gender equality.

Some methodological limitations should be noted. First, the study used a non-probabilistic sample, which may limit the representativeness of the findings despite the sample size and territorial coverage. Second, data were collected exclusively through self-report measures, which may involve response biases such as social desirability. Finally, although appropriate estimation methods for ordinal data were applied, the results should be interpreted with caution given the cross-sectional nature of the design.

### Educational Implications for Non-Sexist Training

From an applied perspective, the results of this study have direct implications for the design of non-sexist educational interventions in higher education. The identification of differentiated profiles of gender stereotypes suggests that students are not a homogeneous group in terms of gender beliefs, which underscores the need for targeted and context-sensitive training strategies.

First, the persistence of stereotypical masculine norms related to emotional restriction and sexual permissiveness highlights the importance of interventions that promote critical reflection on hegemonic masculinity, emotional socialization, and responsibility in intimate relationships. Second, the shared endorsement of feminine caregiving stereotypes by both men and women suggests that non-sexist training should not focus exclusively on male attitudes, but also address the normalization of gendered care expectations among women themselves.

Finally, the presence of moral norms that regulate women’s sexual behavior points to the relevance of incorporating discussions on sexual ethics, autonomy, and gendered moral judgments into university curricula. In this sense, the validated instrument used in this study may serve as a diagnostic tool to identify prevailing belief patterns and to evaluate the impact of educational initiatives aimed at promoting more equitable and non-sexist relationships in higher education contexts.

## Figures and Tables

**Table 1:** Sociodemographic characteristics of the participants (*n* = 672).

Characteristic	%
Sex	
Men	42.7
Women	57.3
Zone	
North	16.3
Center	55.6
South	24.4
Austral South	3.7
Socioeconomic Level	
Low	1.6
Lower Middle	21.6
Middle	60.3
Upper Middle	15.5
High	1

**Table 2:** Factor structure of the scale according to EFA results (n = 336).

Item	Stereotypical Masculine Roles	Stereotypical Feminine Roles	Traditional Roles for Women and Men
1. A real man does NOT show his feelings	0.864		
2. A real man does NOT show his weaknesses	0.821		
3. A man needs several sexual partners	0.997		
4. A man is more rational than a woman	0.661		
5. A family functions better if the man sets the rules	0.769		
6. A man is unfaithful by nature	0.523		
7. A mother is more affectionate than a father		0.666	
8. A woman has greater emotional strength than a man		0.859	
9. Women have a greater ability to care for the sick		0.749	
10. Sons and daughters are better raised by a mother than by a father		0.739	
11. A man is more aggressive than a woman		0.781	
12. A man is less sensitive than a woman		0.550	
13. A woman may achieve fulfillment when she becomes a mother			0.598
14. A good woman should take care of her partner			0.463
15. A woman should protect her family			0.676
16. Infidelity is unforgivable in a woman			0.793
17. A man is more skilled than a woman at flirting			0.480
18. Women have innate abilities for domestic work			0.502

**Table 3:** Fit indices for the factorial models with the confirmation sample (n = 336).

Model	χ^2^ S-B	df	χ^2^/df	TLI	CFI	SRMR	RMSEA (90% CI)
Two correlated factors	2405.800	153	15.72	0.93	0.94	0.100	0.162 (0.154–0.175)
Three correlated factors (EFA results)	638.117	136	4.69	0.99	0.99	0.049	0.078 (0.068–0.089)
Four correlated factors	1510.79	129	11.71	0.95	0.96	0.073	0.129 (0.121–0.134)

**Table 4:** Sex differences in the endorsement of stereotyped roles (n = 672).

Dimension	Women (n = 385) M (SD)	Men (n = 287) M (SD)	Mann–Whitney U Test Z (*p* Value)
Stereotypical Masculine Roles	1.23 (0.32)	1.49 (0.57)	−5.726 (0.000)
Stereotypical Feminine Roles	1.81 (0.62)	2.02 (0.62)	−0.838 (0.402)
Traditional Roles	1.59 (0.56)	2.03 (0.68)	−5.875 (0.000)
Total	1.80 (0.53)	2.01 (0.55)	−4.469 (0.000)

## Data Availability

Data cannot be shared openly but are available on request from the authors.

## References

[B1-behavsci-16-00200] Abarca N., Gormaz R., Leiva F. (2011). Roles vitales y valores tradicionales: Diferencias de género y conservadurismo en estudiantes universitarios chilenos. Revista de Psicología.

[B2-behavsci-16-00200] Araya I., Guizardi M. (2025). Persistent inequalities: A sistematic review on the female presence and gender inequalities in Mexican Academia. CUHSO.

[B3-behavsci-16-00200] Arcos E., Muñoz J., Muñoz P. (2006). Género y universidad: Percepciones de estudiantes y docentes de la Universidad Austral de Chile. Estudios Pedagógicos.

[B4-behavsci-16-00200] Beauvoir S. d. (1949). El segundo sexo.

[B5-behavsci-16-00200] Bem S. L. (1981). Gender schema theory: A cognitive account of sex typing. Psychological Review.

[B6-behavsci-16-00200] Bentler P. M., Chou C. P. (1984). Practical issues in structural modeling. Sociological Methods & Research.

[B7-behavsci-16-00200] Bollen K. A. (1989). Structural equations with latent variables.

[B8-behavsci-16-00200] Bourdieu P. (2000). La dominación masculina.

[B9-behavsci-16-00200] Browne M. W., Cudeck R., Bollen K. A., Long J. S. (1993). Alternative ways of assessing model fit. Testing structural equation models.

[B10-behavsci-16-00200] Butler J. (1990). Gender trouble: Feminism and the subversion of identity.

[B11-behavsci-16-00200] Castillo-Mayén R., Montes-Berges B. (2014a). Analysis of gender stereotypes in a sample of Spanish university students. Psychological Reports.

[B12-behavsci-16-00200] Castillo-Mayén R., Montes-Berges B. (2014b). Influencia de los estereotipos de género en el comportamiento y la percepción de competencia en contextos académicos. Anales de Psicología.

[B13-behavsci-16-00200] Cárdenas M., Lay S., González C., Calderón C., Alegría I. (2010). Validación de la escala de actitudes hacia los roles de Género en una muestra chilena. Revista Latinoamericana de Psicología.

[B14-behavsci-16-00200] Cervilla O., Álvarez-Muelas A., Jimeno Fernández L., Sierra J. C. (2024). Relation between desire and sexual satisfaction in different typologies of adherence to the sexual double standard. Sexuality & Culture.

[B15-behavsci-16-00200] Chadwick-Brown F., Endendijk J. J. (2024). Associations between sexualized media consumption, sexual double standards, and sexual coercion perpetration and victimization in late adolescent sexually active boys and girls from The Netherlands. Archives of Sexual Behavior.

[B16-behavsci-16-00200] Clance P. R., Imes S. A. (1978). The impostor phenomenon in high achieving women: Dynamics and therapeutic intervention. Psychotherapy: Theory, Research & Practice.

[B17-behavsci-16-00200] Cokley K., McClain S., Enciso A., Martínez M. (2018). An examination of the impact of impostor feelings on the mental health of ethnic minority college students. Journal of Multicultural Counseling and Development.

[B18-behavsci-16-00200] Connell R. W. (1995). Masculinities.

[B19-behavsci-16-00200] Cortés M. S. A., Anabalón Y. A., Vega-Román E. (2025). ¿Cambio institucional de género en Educación Superior? Resistencias y perspectivas desde una revisión sistemática de la literatura científica. Revista Fuentes.

[B20-behavsci-16-00200] Crenshaw K. (1989). Demarginalizing the intersection of race and sex: A Black feminist critique of antidiscrimination doctrine. University of Chicago Legal Forum.

[B21-behavsci-16-00200] Díaz-Herrera C., Pino Villalón J. L., López Espinoza M. Á. (2020). Midiendo capital cultural interiorizado: Una escala para educación superior. Revista Pedagogía Universitaria y Didáctica del Derecho.

[B22-behavsci-16-00200] Eagly A. H. (1987). Sex differences in social behavior: A social-role interpretation.

[B23-behavsci-16-00200] Eagly A. H., Karau S. J. (2002). Role congruity theory of prejudice toward female leaders. Psychological Review.

[B24-behavsci-16-00200] Elosua P., Zumbo B. (2008). Coeficientes de Fiabilidad para escalas de respuesta categórica ordenada. Psicothema.

[B25-behavsci-16-00200] Espinoza A. M., Albornoz N. (2023). Sexismo en Educación Superior: ¿Cómo se Reproduce la Inequidad de Género en el contexto Universitario?. Psykhe (Santiago).

[B26-behavsci-16-00200] Farías C., Cuello P. (2018). Juventud, identidades y género: Representaciones en estudiantes universitarios chilenos. Última Década.

[B27-behavsci-16-00200] Flora D., Curran P. (2004). An empirical evaluation of alternative methods of estimation forconfirmatory factor analysis with ordinal data. Psychological Methods.

[B28-behavsci-16-00200] Foucault M. (1975). Vigilar y castigar: Nacimiento de la prisión.

[B29-behavsci-16-00200] Freiberg A., Lorenzo-Seva U., Ferrando P. (2013). Exploratory factor analysis of ordinal variables using parallel analysis and minimum rank factor analysis. Psicothema.

[B30-behavsci-16-00200] Gilligan C. (1982). In a different voice: Psychological theory and women’s development.

[B31-behavsci-16-00200] Glick P., Fiske S. T. (1996). The ambivalent sexism inventory: Differentiating hostile and benevolent sexism. Journal of Personality and Social Psychology.

[B32-behavsci-16-00200] Gómez-Campos R., Cárdenas M., Rivera A. (2023). Adaptación y validación de la escala de estereotipos de género en estudiantes latinoamericanos. Revista Iberoamericana de Diagnóstico y Evaluación Psicológica.

[B33-behavsci-16-00200] Guillén Y., Gutiérrez R., González-Sodis J. (2024). Explorando el enfoque de género en la educación universitaria dominicana: Percepciones y actitudes del profesorado. Revista Espacios.

[B34-behavsci-16-00200] Heilman M. E., Okimoto T. G. (2007). Why are women penalized for success at male tasks? The implied communality deficit. Journal of Applied Psychology.

[B35-behavsci-16-00200] Jerez V. O., Alemán Z. M. (2024). La construcción social de la feminidad y masculinidad como expresiones de la diversidad de géneros en adolescentes de escuelas secundarias. Revista Temas de Mujeres.

[B36-behavsci-16-00200] Kimmel M. (2006). Manhood in America: A cultural history.

[B37-behavsci-16-00200] Lagarde M. (1990). Los cautiverios de las mujeres: Madresposas, monjas, putas, presas y locas.

[B38-behavsci-16-00200] Lamas M. (2000). Cuerpo, diferencia y género.

[B39-behavsci-16-00200] Levant R. F., Wong Y. J. (2017). The psychology of men and masculinities.

[B40-behavsci-16-00200] Lloret-Segura S., Ferreres-Traver A., Hernández-Baeza A., Tomás-Marco I. (2014). El análisis factorial exploratorio de los ítems: Una guía práctica, revisada y actualizada. Anales dePsicología.

[B41-behavsci-16-00200] Lorenzo-Seva U., Ferrando P. (2006). FACTOR: A computer program to fit the exploratoryfactor analysis model. Behavior Research Methods.

[B42-behavsci-16-00200] López-Sáez M., Morales J. F., Lisbona A. (2008). Evolution of gender stereotypes in Spain: Traits and roles. The Spanish Journal of Psychology.

[B43-behavsci-16-00200] Merchan M. E. R., Rendon B. B. (2025). La violencia de género en entornos de la educación superior. Un análisis puntual en la Universidad Técnica de Machala. Ciencia Latina Revista Científica Multidisciplinar.

[B44-behavsci-16-00200] Muñoz-Muñoz D. F., Zambrano-Guerrero C. A. (2024). Roles de género en docentes universitarios: Estudio descriptivo.

[B45-behavsci-16-00200] Navarro C. M. G., Zaragoza C. M. M. (2024). Socialización de género: Identidades, género y otras formas de estratificación social. La Razón Histórica: Revista Hispanoamericana de Historia de las Ideas Políticas y Sociales.

[B46-behavsci-16-00200] Nunnally J. C., Bernstein I. H. (1995). Psychometric theory.

[B47-behavsci-16-00200] Pérez-García R., Pérez-García M., Pérez-García D. (2025). El fundamento simbólico de la violencia de género en el ámbito laboral sanitario. Revista Española de Salud Pública.

[B48-behavsci-16-00200] Pérez Sánchez B., Concha-Salgado A., Fernández-Suárez A., Juarros-Basterretxea J., Rodríguez-Díaz F. J. (2021). La Escala de Actitudes de Roles de Género (GRAS) como una alternativa para la crisis en la medición de actitudes hacia los roles de género en América Latina: Un estudio en universitarios chilenos. Anales de Psicología.

[B49-behavsci-16-00200] Polanco Cerón N., Morrison R. (2024). La ocupación como reproductora del género: Una aproximación a la masculinidad hegemónica. Cadernos Brasileiros de Terapia Ocupacional.

[B50-behavsci-16-00200] R Core Team (2025). R: A language and environment for statistical computing, *Version 4.1*.

[B51-behavsci-16-00200] Robinovich J., Ossa X., Baeza B., Krumeich A., van der Borne B. (2018). Embodiment of social roles and thinness as a form of capital: A qualitative approach towards understanding female obesity disparities in Chile. Social Science & Medicine.

[B52-behavsci-16-00200] Rosseel Y. (2012). Lavaan: An R package for structural equation modeling. Journal of Statistical Software.

[B53-behavsci-16-00200] Rudman L. A., Phelan J. E. (2008). Backlash effects for disconfirming gender stereotypes in organizations. Research in Organizational Behavior.

[B54-behavsci-16-00200] Saldívar-Garduño A., Díaz-Loving R., Reyes-Ruiz N., Armenta-Hurtarte C., López-Rosales F., Moreno-López M., Domínguez-Guedea M. (2015a). Roles de género y diversidad: Validación de una escala en varios contextos culturales. Acta de Investigación Psicológica.

[B55-behavsci-16-00200] Saldívar-Garduño A., Fernández M., Rivera G. (2015b). Escala de Actitudes hacia los Roles de Género (EARG): Propiedades psicométricas en población mexicana. Revista Mexicana de Psicología.

[B56-behavsci-16-00200] Scott J. W. (1990). Gender: A useful category of historical analysis. American Historical Review.

[B57-behavsci-16-00200] Sobarzo M. S., Salinas P. R., Vera M. A. (2025). Percepciones sobre el enfoque de género, la mirada de los profesores y estudiantes en educación superior: ¿Nos acercamos hacia la equidad?. Revista Chilena de Educación Científica.

[B58-behavsci-16-00200] Timmerman M. E., Lorenzo-Seva U. (2011). Dimensionality assessment of ordered polytomous items with parallel analysis. Psychological Methods.

[B59-behavsci-16-00200] Umaña-Taylor A., Yazedjian A., Bámaca-Gómez M. (2004). Developing the Ethnic Identity Scale using Eriksonian and social identity perspectives. Identity: An International Journal of Theory and Research.

[B60-behavsci-16-00200] Umrath B. (2024). Recuperar la dimensión de género de la teoría crítica de la Escuela de Fráncfort: Un análisis feminista. Constelaciones. Revista de Teoría Crítica.

[B61-behavsci-16-00200] UNESCO (2017). Cracking the code: Girls’ and women’s education in STEM.

[B62-behavsci-16-00200] Yetter G., Foutch V. (2013). Investigation of the structural invariance of the ethnic identity scale with native American youth. Cultural Diversity and Ethnic Minority Psychology.

[B63-behavsci-16-00200] Yoon E. (2011). Measuring ethnic identity in the ethnic identity scale and the multigroup ethnic identity measure–revised. Cultural Diversity and Ethnic Minority Psychology.

